# Relationship between adherence to diet, glycemic control and cardiovascular risk factors in patients with type 1 diabetes: a nationwide survey in Brazil

**DOI:** 10.1186/1475-2891-13-19

**Published:** 2014-03-07

**Authors:** Kariane A K Davison, Carlos A Negrato, Roberta Cobas, Alessandra Matheus, Lucianne Tannus, Catia S Palma, Leticia Japiassu, Joao R I Carneiro, Melanie Rodacki, Lenita Zajdenverg, Neuza B C Araújo, Marilena M Cordeiro, Jorge Luiz Luescher, Renata S Berardo, Marcia Nery, Catarina Cani, Maria do Carmo A Marques, Luiz Eduardo Calliari, Renata M Noronha, Thais D Manna, Roberta Savoldelli, Fernanda G Penha, Milton C Foss, Maria Cristina Foss-Freitas, Maria de Fatima Guedes, Sergio A Dib, Patricia Dualib, Saulo C Silva, Janice Sepúlveda, Emerson Sampaio, Rosangela R Rea, Ana Cristina R A Faria, Balduino Tschiedel, Suzana Lavigne, Gustavo A Cardozo, Antonio C Pires, Fernando C Robles, Mirela Azevedo, Luis Henrique Canani, Alessandra T Zucatti, Marisa H C Coral, Daniela A Pereira, Luiz Antonio Araujo, Hermelinda C Pedrosa, Monica Tolentino, Flaviene A Prado, Nelson Rassi, Leticia B Araujo, Reine M C Fonseca, Alexis D Guedes, Odelisa S Mattos, Manuel Faria, Rossana Azulay, Adriana C Forti, Cristina F S Façanha, Renan Montenegro Jr, Ana Paula Montenegro, Naira H Melo, Karla F Rezende, Alberto Ramos, João S Felicio, Flavia M Santos, Deborah L Jezini, Marilia B Gomes

**Affiliations:** 1 Bauru’s Diabetics Association, Department of Internal Medicine, Bauru, São Paulo, Brazil; 2 Department of Internal Medicine, Diabetes Unit, State University Hospital of Rio de Janeiro, Rio de Janeiro, Brazil; 3 Federal University Hospital of Rio de Janeiro, Rio de Janeiro, Brazil; 4 General Hospital of Bonsucesso, Rio de Janeiro, Brazil; 5 University Hospital Clementino Fraga Filho-Children Institute Martagão Teixeira, Rio de Janeiro, Brazil; 6 Diabetes Unit, University Hospital of São Paulo, Sao Paulo, Brazil; 7 Pediatric Unit of Endocrinology-Santa Casa Hospital, São Paulo, Brazil; 8 Children Institute of Endocrinology, University Hospital of São Paulo, São Paulo, Brazil; 9 Ribeirão Preto Medical School of São Paulo University, Sao Paulo, Brazil; 10 Diabetes Unit, Federal University of São Paulo State, São Paulo, Brazil; 11 Endocrinology Unit, Hospital of Santa Casa of Belo Horizonte, Minas Gerais, Brazil; 12 Diabetes Unit, State University Hospital of Londrina, Paraná, Brazil; 13 Clinical Hospital of the Federal University of Paraná, Paraná, Brazil; 14 Institute of Diabetic Children, Rio Grande Sul, Brazil; 15 Department of Internal Medicine, Medical School, State University of São José do Rio Preto, São Paulo, Brazil; 16 Clinical Hospital of Porto Alegre, Rio Grande do Sul, Brazil; 17 University Hospital of Santa Catarina, Santa Catarina, Brazil; 18 Endocrinology and Diabetes Institute of Joinville, Santa Catarina, Brazil; 19 Regional Hospital of Taguatinga, Brasília, Brazil; 20 General Hospital of Goiânia, Goiás, Brazil; 21 Diabetes and Endocrinology Center of Bahia, Bahia, Brazil; 22 Federal University of Maranhão, Maranhão, Brazil; 23 Diabetes and Hypertension Center of Ceará, Ceará, Brazil; 24 Federal University of Ceará, Ceará, Brazil; 25 Federal University of Sergipe, Sergipe, Brazil; 26 Federal University Hospital of Campina Grande, Paraíba, Brazil; 27 Federal University Hospital of Pará, Pará, Brazil; 28 Getúlio Vargas University Hospital of Amazonas, Adriano Jorge Hospital, Manaus, Amazonas, Brazil; 29 Rua Saint Martin 27-07, Vila Universitária, Bauru, São Paulo 17012-433, Brazil

**Keywords:** Type 1 diabetes, Glycemic control, Dietitian, Diet, Diabetes care, Adherence to diet

## Abstract

**Background:**

To determine the relationship between adherence to the diet reported by patients with type 1 diabetes under routine clinical care in Brazil, and demographic, socioeconomic status, glycemic control and cardiovascular risk factors.

**Methods:**

This was a cross-sectional, multicenter study conducted between December 2008 and December 2010 in 28 public clinics in 20 Brazilian cities. The data was obtained from 3,180 patients, aged 22 ± 11.8 years (56.3% females, 57.4% Caucasians and 43.6% non-Caucasians). The mean time since diabetes diagnosis was 11.7 ± 8.1 years.

**Results:**

Overall, 1,722 (54.2%) of the patients reported to be adherent to the diet without difference in gender, duration of diabetes and socioeconomic status. Patients who reported adherence to the diet had lower BMI, HbA1c, triglycerides, LDL-cholesterol, non HDL-cholesterol and diastolic blood pressure and had more HbA1c at goal, performed more frequently self-monitoring of blood glucose (p < 0.001), and reported less difficulties to follow specific schedules of diet plans (p < 0.001). Less patients who reported to be adherent were obese or overweight (p = 0.005). The quantity of food and time schedule of the meals were the most frequent complaints. Logistic regression analysis showed that ethnicity, (Caucasians, (OR 1.26 [1.09-1.47]), number of medical clinical visits in the last year (OR 1.10 [1.06-1.15]), carbohydrate counting, (OR 2.22 [1.49-3.30]) and diets recommended by diabetes societies’, (OR 1.57 [1.02-2.41]) were related to greater patients’ adherence (p < 0.05) and age, [adolescents (OR 0.60 [0.50-0.72]), high BMI (OR 0.58 [0.94-0.98]) and smoking (OR 0.58 [0.41-0.84]) with poor patients’ adherence (p < 0.01).

**Conclusions:**

Our results suggest that it is necessary to rethink medical nutrition therapy in order to help patients to overcome barriers that impair an optimized adherence to the diet.

## Introduction

The treatment of diabetes should start with non-pharmacological therapies such as lifestyle interventions. A healthy lifestyle with regular physical activity and healthy eating are very important tools in reaching and maintaining an adequate glycemic control in patients with type 1 diabetes (T1D) [1].

Health care professionals are frequently challenged with the task of motivating patients to follow dietary and exercise guidelines and take insulin injections to improve their diabetes control and thereby slow or avoid the occurrence of diabetes-related acute and chronic complications. Lifestyle modification is an integral part of T1D management. Patients with T1D, because of a universal need for insulin, must learn to count or closely estimate the amount of carbohydrate they consume to help regulate their blood glucose levels and adjust their insulin doses. Failure to do so can lead to hyperglycemia or hypoglycemia [1].

Medical nutrition therapy (MNT) is important at all levels of diabetes care. MNT is also an integral component of diabetes self-management education and training. The first nutrition priority for individuals requiring insulin therapy is to change their lifestyle in order to incorporate an insulin regimen into their preferred diet and exercise routines. With the many insulin options now available, an appropriate insulin regimen can usually be developed to conform to an individual’s preferred meal routine, food choices and physical activity pattern [2].

In addition, the guidelines recommend reaching an optimal glycemic control avoiding the development of overweight or obesity as well as hypoglycemia and diabetes-related comorbidities (hypertension and dyslipidemia) and cardiovascular diseases [3].

The diet regimen for T1D is complex. Studies conducted in order to investigate the diet adherence of children and adolescents with T1D have found rates of dietary adherence ranging from 21% to 56% based on self-reported adherence rates [4] and rather poor adherence to nutritional recommendations in adults with T1D [5,6].

The aim of this study was to determine the relationship between the adherence to the diet reported by the patient and demographic, socioeconomic status, glycemic control and cardiovascular risk factors, in patients with T1D under routine clinical care in Brazil.

## Research design and methods

This was a retrospective observational, cross-sectional, multicenter study conducted between December 2008 and December 2010 in 28 secondary and tertiary care public clinics. These clinics were located in 20 cities within four Brazilian geographic regions (north/northeast, midwest, southeast and south). The methodology has been described previously [7]. Briefly, all patients received health care from the National Brazilian Health Care System (NBHCS). Each clinic provided data from at least 50 T1D outpatients that regularly attended this clinic. All patients were treated by an endocrinologist in secondary or tertiary care settings. The inclusion criteria included T1D patients diagnosed by a physician based on a typical clinical presentation including variable degrees of weight loss, polyuria, polydipsia and polyphagia, as well as the need of using insulin continuously since the diagnosis. Appendix lists each local center’s ethics committee approval of the study. Each center had a coordinator who was trained to analyze the data that were obtained from the medical charts.

Our sample size was of 3,591 patients; however, this study was comprised of only patients with at least one year of medical follow-up at each respective center that was a total of 3,180 patients (88.5%). All patients were diagnosed with T1D between 1960 and 2010. Patients younger than 13 years old were considered to be children, patients between 13 and 19 years old were classified as adolescents, and patients older than 19 were considered to be adults according to the American Diabetes Association criteria (ADA) [2].

The following variables were assessed by a questionnaire applied during a clinical visit: current age, age at diagnosis, diabetes duration, height (m), weight (kg), treatment modalities for diabetes or diabetes-related comorbidities, frequency of self-monitoring of blood glucose (SMBG) and smoking status. The questionnaire included also self-reported questions related to nutritional factors associated with diet in daily clinical practice such as if the patient followed any prescribed diet, the specific health care professional that prescribed the diet, how adherent to the reported diet patients were (it is of note that adherence was defined as following at least 80% of the time of the reported diet), type of reported diet, main difficulties found to follow the diet, presence of comorbidities, self-reported frequency of severe hypoglycemia and hospitalization because of either diabetes ketoacidosis or hyperglycemia. The levels of HbA1c, fasting plasma glucose (FPG), total cholesterol, low-density lipoprotein (LDL) cholesterol, high-density lipoprotein (HDL) cholesterol, and triglycerides measured during the last clinical visit were obtained from the patients’ medical records. Within one year of the study assessment, the patients with a diabetes duration greater than or equal to five years were screened for chronic diabetes-related complications: retinopathy (classified as absent, nonproliferative, or proliferative; by fundoscopy); clinical nephropathy (according to ADA recommendations [8]; macrovascular diseases (classified as clinical coronary artery disease, stroke, and peripheral vascular disease); and foot pathologies.

The following ADA goals for adequate metabolic and clinical control [8] were adopted by the Brazilian Type 1 Diabetes Study Group (BrazDiab1SG). Good glycemic control (HbA1c at goal) was defined as HbA1c levels of < 58 mmol/mol (7.5%) for T1D patients between 13 and 19 years old; < 64 mmol/mol (8%) for patients between 6 and 12 years old; between 58 mmol/mol (7.5%) and 69 mmol/mol (8.5%) for patients < 6 years old; and <53 mmol/mol (7%) for adult T1D patients (8). Poor glycemic control was defined as HbA1c levels higher than 75 mmol/mol (9%).

The body mass index (BMI) was determined by dividing an individual’s weight (kg) by the square of the height (m^2^). In adults, being overweight was defined as having a BMI of ≥ 25 kg/m^2^, and obesity was defined as a BMI of ≥ 30 kg/m^2^[9]. In children and adolescents, overweight was defined as a BMI of ≥ 85th percentile for age and gender, and obesity was defined as a BMI of ≥ 95th percentile for age and gender [10].

In 2,765 patients (87.1%), HbA1c was measured using methods certified by the National Glycohemoglobin Standardization Program (NGSP): high- performance liquid chromatography in 1,512 patients (54.7%) and turbidimetry in 1,253 patients (45.3%). HbA1c levels determined using methods that were not certified by the NGSP and patients with missing data were excluded from the analyses (n = 411, 12.9%).

### Sample calculation and socioeconomic status definition

A detailed description of how the study sample was selected has been described previously [9]. Briefly, the study aimed to represent the distribution of T1D cases across each geographic region of Brazil estimated according to the population distribution reported by the 2000 Brazilian Institute of Geography and Statistics Census (IBGE) [11] combined with national estimates of diabetes prevalence derived from a survey conducted in 1988, to determine the minimum number of patients that should be studied in each region [12]. Each region enrolled more than 95% of the estimated number of patients in this study. Socioeconomic status were defined according to the Brazilian Economic Classification Criteria which also considers education level, which is categorized as illiterate/incomplete primary education, complete primary education/incomplete secondary education, complete secondary education/incomplete high school, complete high school/some college and complete college education [13]. For this analysis, the following classes of economic status were considered: high, middle, low and very low.

### Statistical analysis

The data are presented as the mean (± SD) or the median (minimum-maximum) for continuous variables and as numbers (relative frequencies) for discrete variables. Comparisons between independent continuous variables were performed using independent, two-sided t-tests, linear association or ANOVA with Bonferroni correction, as indicated. Two-sided Z-tests were used for comparisons between discrete variables with a normal approximation to the binomial distribution. A multiple logistic regression was performed with adherence to the prescribed diet (yes/no) as dependent variable. The following independent variables were included: ethnicity (Caucasian or non-Caucasian established by medical charts or self-report); age (categorized in three groups: 0–12.9, 13.0-18.9 and ≥19.0 years); gender; type of reported diet, duration of diabetes, level of care (tertiary or secondary), follow-up time in each center, smoking status, BMI, number of medical clinical visits in the previous year and socioeconomic status. For this analysis, the Nagelkerke R-squared was also calculated. The analyses were performed using SPSS version 16.0 (Statistical Package for the Social Sciences, Chicago, Illinois). Odds ratios with 95% CI were expressed as indicated. A two- sided p value of less than 0.05 was considered significant. When using multiple statistical tests, the p-values were adjusted using the Bonferroni correction.

## Results

### Overview of adherence to the prescribed diet and participant demographics, economic status, level of care and insulin treatment modalities

Demographic data are detailed in Table 1. Overall, 1,722 (54%) of the patients reported adherence to the prescribed diet, without difference according to gender, mean age, age at diagnosis of diabetes, duration of diabetes, economic status, geographical region of the country, time of follow-up at each respective center and level of care. More Caucasian patients reported adherence to the diet than non-Caucasian patients (p = 0.001). The comparison between adolescents and adults showed that the former reported less adherence to the diet (p < 0.001). These data are described in Table 2.

**Table 1:** Clinical and demographic data of the studied population

**Variable**	
**N**	3,180
**Female, n (%) Age, y**	1,791 (56.3)
**Age range, y, n (%)**	22 ± 11.8
0-4.9	51 (1.6)
5-9.9	308 (9.7)
10-14.9	604 (19)
15-29.9	1,471 (46.3)
30 or older	746 (23.5)
**Ethnicity, n (%)**	
Caucasian	1,824 (57.4)
Non-Caucasian*	1,356 (42.6)
Economic status**	
High	229 (7.2)
Medium	710 (22.3)
Low	1,052 (33.1)
Very low	1,102 (34.7)
**Level of care n (%)**	
Secondary	897 (28.2)
Tertiary	2,283 (71.8)
**Duration of diabetes, y**	10.3 ± 8.04

y = year; data are presented as number (percentage) or mean ± SD.

*African-Brazilians, Mulattos, Asians, and Native Indians. **Missing data from 87 participants.

**Table 2:** Demographic, clinical and nutrition management in patients with type 1 diabetes according to adherence to the prescribed diet

**Variable**	Adherence *		***P-*** **value**
	**Yes (%)**	**No (%)**	
**N**	1,722 (54.2)	1,458 (45.8)	-
**Demographic and social data**			
**Age**	22.1 ± 12.3	21.8 ± 11.0	0.5
**Age range, y, n (%)**			<0.001
**0-12,9**	415 (24.1)	251 (17.2)	
**13-18,9**	374 (21.7)	430 (29.9)	
**≥19**	933 (54.2)	822 (53.3)	
**Age at diagnosis of diabetes**	11.6 ± 8.3	11.6 ± 7.7	0.5
**Female, n (%)**	962 (55.9)	829 (56.9)	0.4
**Duration of diabetes**	10.4 ± 8.3	10.2 ± 7.7	0.9
Ethnicity**			
**Caucasian**	1,032 (59.9)	792 (43.4)	0.001
**Non-caucasian**	690 (50.9)	666 (49.1)	
Economic status, n (%)***			0.08
**High**	134 (8.1)	95 (6.6)	
**Medium**	397 (23.9)	313 (21.9)	
**Low**	570 (34.3)	482 (33.7)	
**Very Low**	563 (33.8)	539 (37.7)	
**Geographical region**			0.5
**North/Northeast**	525 (30.5)	413 (28.3)	
**Southeast**	703 (40.8)	607 (41.6)	
**South**	389 (22.6)	338 (23.2)	
**Midwest**	105 (6.1)	100 (6.9)	
**Medical nutrition therapy**			
**Appointment with dietitian, y (%)**	1,288 (75.0)	979 (43.2)	<0.001
**Diet prescriptor**			0.07
**Dietitian**	1,188 (56.4)	918 (43.6)	
**Endocrinologist**	435 (52.8)	389 (47.2)	
**Difficulties to follow the prescribed diet, y(%)**			
**Avoiding sugar and sweets**	620 (36.0)	743 (51.0)	<0.001
**Eating vegetables and fruits**	308 (17.9)	339 (23.3)	<0.001
**Quantity of prescribed foods**	811 (47.1)	971 (66.6)	<0.001
**Schedule time of the meals**	778 (45.2)	881 (60.4)	<0.001
**Understanding foods substitution lists**	350 (20.4)	476 (32.7)	<0.001
**Glycemic control**			
**HbA1c (%)**	8.8 ± 2.1	9.9 ± 2.4	<0.001
**HbA1c (mmol/mol)**	73.5 ± 23.5	84.3 ± 26.8	
**HbA1c at goal, n (%) **^**##**^	317 (21.1)	145 (11.5)	<0.001
**HbA1c ≥ 9.0%, n (%)**^**###**^	740 (43.0)	799 (47.2)	0.2
**Hospitalization, y (%) **^**#**^	176 (48.2)	185 (51.2)	0.03
**Severe hypoglycemia, y (%)**	179 (16.09)	141 (16.9)	0.5
SMBG **** y, (%)	1,377 (91.6)	1,106 (87.5)	<0.001
**SMBG (n)**	3.5 ± 1.7	3.1 ± 1.6	<0.001
**Number of medical clinical visits (previous year)**	4.3 ± 1.6	3.9 ± 1.7	0.001
**Cardiovascular risk factors**			
**BMI**	21.6 ± 4.2	22.8 ± 4.3	0.001
**Overweight or obesity, n (%)**^**####**^	446 (29.8)	437 (34.7)	0.005
**sBP (mmHg)**	111.7 ± 17.5	112.2 ± 16.7	0.4
**dBP (mmHg)**	71.3 ± 11.4	72.4 ± 11.4	0.01
**Cholesterol (mg/dl)**	167.1 ± 39.6	174.7 ± 43.3	<0.001
**Triglycerides (mg/dl)**	85.9 ± 59.4	99.3 ± 78.5	<0.001
**HDL-cholesterol (mg/dl)**	53.5 ± 14.8	52.3 ± 14.4	0.04
**LDL-cholesterol (mg/dl)**	97.2 ± 31.8	103.3 ± 34.4	<0.001
**Non-HDL-cholesterol (mg/dl)**	113.4 ± 36.4	122.4 ± 42.6	<0.001
**Current smoker, y (%)**	56 (3.3)	81 (5.6)	<0.001

*The data are presented as number (percentage) or mean ± SD.

**African-Brazilian, Mulatto, Asian, or Native Indian.

***Missing data: 87 patients.

****SMBG: self monitoring blood glucose.

^#^Hospitalization for hyperglycemia or ketoacidosis.

^##^ HbA1c at goal was defined as: as HbA1c levels of < 58 mmol/mol (7.5%) for T1D patients between 13 and 19 years old; < 64 mmol/mol (8%) for patients between 6 and 12 years old; between 58 mmol/mol (7.5%) and 69 mmol/mol (8.5%) for patients < 6 years old; and <53 mmol/mol (7%) for adult T1D patients.

^###^HbA1c levels higher than 75 mmol/mol (9%) was defined as poor glycemic control.

^####^Overweight and obesity were considered together.

An association was also observed between insulin regimens and a reported adherence to the prescribed diet as follows: among the patients treated by conventional therapy (CT) (intermediate human insulin one to two injections daily), 163 (51.6%) reported to be adherent to the diet; among those on intensive therapy (IT) 761(50.7%) of the patients treated by one or two injections of intermediate human insulin plus human regular insulin; 324 (52.7%) of the patients treated by one or two insulin injections of intermediate human insulin plus short-acting insulin- analogues; 363 (64.0%) of the patients treated by basal-bolus (one or two insulin injections of long-acting insulin-analogues plus short-acting insulin-analogues or long-acting insulin-analogues plus regular insulin); and 29 (76.3%) of the patients treated by basal-bolus with continuous subcutaneous insulin infusion (CSII) reported to be adherent to the diet. The adherence was greater in patients using basal-bolus (one or two insulin injections of long-acting insulin-analogues plus short-acting insulin-analogues or long-acting insulin-analogues plus regular insulin and CSII in comparison to each of the other groups (p < 0.001). No difference was found between both insulin regimens.

### Overview of medical nutrition therapy and adherence to the prescribed diet and its determinants

More patients who reported adherence to the diet had had an appointment with a dietitian in the previous year in comparison to those patients that did not have an appointment. The adherence to diet was not related to who prescribed the diet, either a dietitian or an endocrinologist.

The majority of the patients 1,546 (48.6%) followed a diet avoiding only sweets and sugar, 397 (12.5%) followed a diet according to Brazilian Diabetes Society (BDS) [14] and the ADA 2008 recommendations [2], 967 (30.4%) followed a diet of carbohydrate counting, 155 (4.9%) a diet according to foods’ glycemic index and 115 (3.6%) other types of diet.

An association was noted between adherence and type of diet as follows: 626 (36.4%) of the patients who reported a diet of carbohydrate counting, 224 (13.0%) of the patients who reported a diet according to BDS and ADA’s 2008 recommendations; 80 (4.6%) of the patients who reported a diet according to foods’ glycemic index; 741 (43.0%) of the patients who reported a diet avoiding only sweets and sugar; and 51 (3.0%) of the patients who reported other types of diet, (p < 0.001).

Patients who reported adherence to the diet reported less difficulties to follow the diet compared to those that were not adherent (p < 0.001). These data are described in Table 2.

Using multivariate analysis and adjusting for other variables, the probability of a given patient be adherent to the prescribed diet was of 6.7%. The independent variables associated with adherence were ethnicity, age, BMI, number of medical clinical visits in the previous year, smoking status, carbohydrate counting and diets recommended by BDS and ADA’s. The adjusted model is shown in Table 3.

**Table 3:** **Final adjusted logistic regression model with adherence to the prescribed diet as dependent variable**^#^

			**Adjusted**
	**N**	**OR (95% CI)**	***P-*** **value**
**Ethnicity**			*reference*
NonCaucasians*	1,318		
Caucasians	1,762	1.26 (1.09-1.47)	0,003
**Type of *reported *** **diet**			
Other types of diet**	115	1	*reference*
Avoiding only sweets and sugar	1,498	1,12 (0.76 - 1.66)	0.53
Regular diet***	379	1.57 (1.02-2.41)	0.03
Carbohydrate counting	938	2.22 (1.49- 3.30)	<0.001
Glycemic index	150	1.21 (0.73-2.00)	0.44
**Age**			
≥19 years	1,702	1	reference
13-18.9 years	802	0.60 (0.50-0.72)	<0.001
0-12.9 years	576	0.94 (0.74-1.20)	0.66
**BMI**	3,080	0.58 (0.94-0.98)	0.001
**Number of medical clinical visits (previous year)**	3,080	1.10 (1.06-1.15)	<0.001
(previous year)			
**Current smoker, y**	135	0.58 (0.41- 0.84)	0.004

^#^Missing data of 100 patients.

*African-Brazilians, Mulattos, Asians, and Native Indians.

**Other type of diet included diets like gluten free, vegetarian and hypocaloric.

***Regular diet: diet according to ADA and BDS statement (references [2,15]).

### Overview of adherence to the prescribed diet and glycemic control

A lower mean of HbA1c levels was observed in patients who reported adherence to the diet in comparison to the other group. More patients who reported adherence to the diet reached the target of HbA1c, and performed SMBG more frequently. Less patients which reported adherence to the diet had hospitalization because ketoacidosis or hyperglycemia. Severe hypoglycemia was not associated with adherence. These data are described in Table 2.

### Overview of adherence to the prescribed diet and cardiovascular risk factors

Being overweight or obese was observed in 990 patients (31.3%). Less patients who reported adherence to the prescribed diet were overweight or obese and were current smokers in comparison to the other group. Patients who reported adherence to the diet had lower BMI, diastolic blood pressure, total cholesterol, triglycerides, LDL-cholesterol, non HDL-cholesterol and higher HDL-cholesterol than the other group. These data are described in Table 2.

## Discussion

The evaluation of adherence to the prescribed diet in Brazilian patients with T1D revealed that approximately 54.2% of them were adherent to the reported diet. The majority of these patients were Caucasians, had gone to more medical clinical visits in the previous year and were under carbohydrate counting and diets recommended by diabetes societies’ guidelines and using more complex schedules of insulin therapy. Moreover, adherence to the diet was associated with better glycemic and cardiovascular risk factors control including lower rates of overweight or obesity and healthier lifestyle habits such as non-smoking. The most frequent difficulties to follow the diet reported by the patients were the quantity of prescribed food and the eating time patterns.

A systematic review of 23 manuscripts on dietary adherence in youth with T1D showed rates of adherence to eating behaviors ranging from 21% to 95% and studies examining the contents of macronutrients and dietary recommendations revealed higher intakes of fat and saturated fat and lower than recommended intakes of fruits, vegetables, and whole grains [15]. The same was found among adults with T1D [5,6].

Several studies have shown problems with dietary adherence in patients with T1D [4-6,16]; in our study we have found that the adherence to the diet was less significantly found in adolescents than in adults [16,17]. Some studies have established that current age, gender, and diabetes duration are crucial non-modifiable risk factors for diabetes poor management and suggest that adolescents who are older, whose disease duration is longer are more likely to have problems with diabetes self-care [18-20]. Previous evidence on a larger multiethnic sample of youth with T1D suggest that both progressive loss of beta cell function and difficulties in maintaining a long lasting motivation for the intensive daily diabetes care patterns required for an optimal glucose control are risk factors for poor glucose control and decreased adherence to diabetes management tasks, which often occur during the adolescence [19,20].

The adherence to the diet was higher in patients who reported an appointment with a dietitian in the previous year compared with patients who had not [21,22]. It is well established that appointments with a dietitian is beneficial to the management of diabetes in youth and improve their eating habits and glycemic control [17].

An association between the type of reported diet and adherence showed that carbohydrate counting [23] was frequently performed, following the recommendations of the BDS [14] and ADA [2]. The interest in carbohydrate counting is increasing since its results are effective and allows greater flexibility in foods choice [23,24]. Although controversial, the glycemic index diet [25] was also reported by some patients. The data concerning those diet avoiding only sweets and sugar is consistent with a study that has found differences in perceptions of healthy eating versus perceptions of eating practices that are good for diabetes management. Specifically, foods high in fat but low in carbohydrate were commonly reported as being good for diabetes management [26]. Another study has found parents and youth that classified fruits as “unhealthy” foods because they can lead to higher postprandial glucose levels. They identified “healthy” vs “unhealthy” foods based on their effect on glycemic control [25]. There are many barriers that prevent T1D patients adhering to the treatment and to the diet more specifically. For adults with T1D twelve types of problematic situations have been identified: negative emotions, resisting temptation, eating out, feeling deprived, time pressure, temptation to relapse, planning, competing priorities, social events, family support, food refusal and absence of friends’ support [27]. In young adults with an average age of 22 years, family conflicts, psychological problems, and carbohydrate counting obstacles remain unsolved problems worsening glycemic control [28]. In youth aged 7 to 16 years, the most commonly discussed barrier to healthy eating was the constant and extensive exposure to unhealthy food at and outside home, peer interactions, convenience (preference for prepackaged foods that require no preparation) and consuming fast food and other less healthy meals because of busy schedules and lack of time to prepare the meals [26]. A recent study conducted with adolescents with T1D identified 10 relatively homogeneous categories of obstacles to dietary adherence: being tempted to stop trying; negative emotional eating; facing forbidden foods; peer interpersonal conflict; competing priorities; eating at school; social events and holidays; food cravings; snacking when at home, alone, or bored; and social pressure to eat [29]. The difficulty in understanding the proposed food plans, for example lists of replacements or carbohydrate counting therapy charts [30-32] are problems that are frequently reported.

It is well documented that MNT favors the reduction of glycated hemoglobin levels [14,25,33]. Similar to our data, a study with T1D, aged between 9–14 years showed that adherence to diet was associated with better HbA1c levels and more frequent SMBG. A relationship between modality of insulin injection and diet adherence showed that patients in more complex insulin therapy schedules were more adherent to diet. The same was observed in the study that investigated the association between dietary adherence and glycemic control. The results showed that patients treated with ≥ 4 injections/day or insulin pump were in the highest and middle level of dietary adherence and concluded that greater dietary adherence was associated with lower A1c among youth with type 1 diabetes [34]. Healthy eating habits were also associated with better glycemic control in adolescents [35,36] and adults who have been training flexible intensive insulin treatment combined with more relaxed dietary flexibility and with insulin doses adjustment, have shown to improve both glycemic control and quality of life [23].

Our results are consistent with other studies linking dietary intervention with lower levels of sBP, dBP, triglycerides, HDL and LDL cholesterol [37,38]. The high intake of calories originated from fat and low intake of fibers, fruits and vegetables is a concern given the risk that T1D poses for CVD [20,39-42]. The adherence to the prescribed diet is a contributing factor to the reduction of the cardiovascular risk factors [25] and weight control [36]. Our study has shown that patients who reported being adherent to diet had lower rates of overweight and obesity. A Norwegian study has shown that skipping meals is associated with negative stigma such as being overweight and having a higher intake of added sugar and lower intake of fiber [36]. Close adherence to dietary management has been found to be correlated with better glycemic control in youth with T1D [16,34] and following recommendations for healthy eating may be the best way for preventing or treating comorbid conditions [2].

Multiple injections therapy has been found in several studies to be a main predictor of weight gain, overweight and obesity that are cardiovascular risk factors. This could be explained by increased caloric intake due to the flexibility allowed by intensive insulin treatment [43,44]. This greater flexibility may also increase the opportunity to choose types and amounts of foods, potentially making nutritional education even more important [26]. Our study showed better adherence to diet in non-smoking patients which could be related to a better adherence to behavioral advice (diet, exercise, and smoking cessation) although we did not address adherence to exercise. It is important to emphasize that better adherence to behavioral advice after acute coronary syndrome was associated with a substantially lower risk of recurrent cardiovascular events [45].

We did not find significant difference in poor glycemic control between subjects who did follow or did not follow the diet, probably because the number of patients with an inadequate glycemic control, evaluated by the HbA1c levels was very expressive (almost 47% of the patients had HbA1c levels higher than 9%).

The primary strength of our large sample size is that it represents the diverse, young T1D Brazilian population. Patients included in the study belonged to a wide range of ethnic groups and socioeconomic backgrounds from all of the geographic regions of the country, with a uniform, standardized recruitment protocol in all participating centers.

Finally, our study has also some limitations that must be mentioned. One limitation was the sample characteristics. All patients lived in large cities and were cared for in a public health center by a specialist; thus, patients who rely on primary care facilities and live in rural areas may not have been considered. However, the latter T1D patients are the minority of those receiving treatment in Brazil. Additionally, the recruitment of patients within each center may have led to a selection bias. Also, the data related to nutritional factors were self reported including adherence to diet which was based on information reported on adherence to medication in clinical trials [46]. These data were obtained during an interview conducted by a doctor during a clinical evaluation and were not based in diet diary (work and weekend days). The lack of standardization for evaluation of HbA1c levels which is currently an unsolved problem in our country, lack of control for physical activity and the absence of psychosocial evaluation were other limitations of our study. Family support and patient self-efficacy has been associated with several positive outcomes, including better glycemic control and compliance to perform SMBG but this was not investigated concerning the adherence to a specific diet plan.

In conclusion, despite the advantages of being adherent to the diet upon glycemic and cardiovascular risk factors control, nearly 45% of Brazilian T1D patients did not report adherence to the diet. The diabetes care team must change the approach to the patients and their families reinforcing the importance of the diet in reaching an adequate metabolic control and consequently in avoiding or postponing diabetes-related complications and also helping them to overcome the difficulties they have in following the diet. Our results suggest that it is necessary to rethink medical nutrition therapy in order to help patients to overcome barriers that impair an optimized adherence to the diet.

## Appendix

### Brazilian Type 1 Diabetes Study Group (BrazDiab1SG)

Executive steering committee: Marilia Brito Gomes (chair), Roberta Cobas, Sergio Atala Dib, Carlos Antonio Negrato.

Principal investigators are indicated by an asterisk. Program coordinators are in italics.

**Department of Internal Medicine, Diabetes Unit, State University of Rio de Janeiro, Brazil**: Roberta Cobas*, M.D. (robertacobas@gmail.com), *Alessandra Matheus*, M.D. (alessandramatheus79@yahoo.com), Lucianne Tannus, M.D. (luciannetannus@ig.com.br), Catia Cristina Sousa Palma, M.D. (catiasousapalma@gmail.com), Leticia Japiassu,M.D. (leticiamauricio@gmail.com); Marilia Brito Gomes, M.D. (mariliabgomes@gmail.com), João Regis Ivar Carneiro, M.D. (endoregis@uol.com.br); **Federal University Hospital of Rio de Janeiro**: Melanie Rodacki*, M.D. (mrodacki2001@yahoo.com.br), *Lenita Zajdenverg*, M.D. (lenitazaj@gmail.com); **General Hospital of Bonsucesso**: Neuza Braga Campos de Araújo*, M.D. (russarj@terra.com.br), *Marilena de Menezes Cordeiro*, M.D. (marmecor@gmail.com); **University Hospital Clementino Fraga Filho – Children Institute Martagão Teixeira**: Dr. Jorge Luiz Luescher*, M.D. (luescher_@hotmail.com), *Renata Szundy Berardo*, M.D. (rszundy@br.inter.net); **Diabetes Unit, University Hospital of São Paulo, São Paulo**: Marcia Nery*, M.D. (marcianery@hcnet.usp.br), *Catarina Cani*, M.D. (catarinagcani@gmail.com), Maria do Carmo Arruda-Marques, M.D. (mcarruda@hcnet.usp.br); **Pediatric Unit of Endocrinology - Santa Casa Hospital, São Paulo**: Luiz Eduardo Calliari*, M.D. (calliari.cidep@uol.com.br), *Renata Maria de Noronha*, M.D. (renata_noronha@uol.com.br); **Children Institute of Endocrinology, University Hospital of São Paulo, São Paulo**: Thais Della Manna*, M.D. (thais.manna@icr.usp.br), *Roberta Savoldelli*, M.D. (robysds@hotmail.com), Fernanda Garcia Penha, M.D. (fepenha@ajato.com.br); **Ribeirão Preto Medical School of São Paulo University, Sao Paulo**: Milton Cesar Foss*, M.D. (mcfoss@fmrp.usp.br), *Maria Cristina Foss-Freitas*, M.D. (crisfoss@fmrp.usp.br); **Department of Internal Medicine, Medical School, State University of SãoJosé do Rio Preto**: Antonio Carlos Pires*, M.D. (fpires@terra.com.br), *Fernando Cesar Robles*, M.D. (roblesmed@ig.com.br); **Bauru’s Diabetics Association, Bauru, São Paulo**: Carlos Antonio Negrato* , M.D. (carlosnegrato@uol.com.br), *Maria de Fatima Guedes*, M.D. (tatiguedeses@hotmail.com); **Diabetes Unit, Federal University of São Paulo State, São Paulo**: Sergio Atala Dib*, M.D. (sergio.dib@unifesp.br), *Patricia Dualib*, M.D. (patricia.dualib@uol.com.br); **Endocrinology Unit, Hospital of Santa Casa of Belo Horizonte, Minas Gerais**: Saulo Cavalcanti da Silva*, M.D. (scsendocrino@yahoo.com.br), *Janice Sepúlveda*, M.D. (janicesepulveda@terra.com.br); **Diabetes Unit, State University Hospital of Londrina, Paraná**: *Emerson Sampaio*, M.D. (emersamp@hotmail.com); **Clinical Hospital of the Federal University of Paraná**: Rosangela Roginski Rea*, M.D. (rosangelarea@uol.com.br), *Ana Cristina Ravazzani de Almeida Faria*, M.D. (aravazzani@uol.com.br); **Institute of Diabetic Children, Rio Grande Sul**: Balduino Tschiedel*, M.D. (badutsch@gmail.com), *Suzana Lavigne*, M.D. (suzanalavigne@yahoo.com.br), Gustavo Adolfo Cardozo, M.D. (byguga@hotmail.com); **Clinical Hospital of Porto Alegre, Rio Grande do Sul**: Mirela Azevedo*, M.D. (mirelajobimazevedo@gmail.com), *Luis Henrique Canani*, M.D. (luishenriquecanani@gmail.com), Alessandra Teixeira Zucatti, M.D. (alezucatti@hotmail.com); **University Hospital of Santa Catarina**: Marisa Helena Cesar Coral*, M.D. (marisahcc@uol.com.br), *Daniela Aline Pereira*, M.D. (danialine@yahoo.com); **Endocrinology and Diabetes Institute of Joinville, Santa Catarina**: Luiz Antonio de Araujo*, M.D. (luiz@endoville.com.br); **Regional Hospital of Taguatinga, Brasília**: Hermelinda Cordeiro Pedrosa*, M. D (pedrosa.hc@globo.com), *Monica Tolentino*, M.D. (monicatolentino@uol.com.br); Flaviene Alves Prado, M.D. (secretariadraflaviene@gmail.com); **General Hospital of Goiânia:** Nelson Rassi*, M.D. (nrassi@brturbo.com.br), *Leticia Bretones de Araujo*, M.D. (leticiabretones@yahoo.com.br); **Diabetes and Endocrinology Center of Bahia**: Reine Marie Chaves Fonseca*, M.D. (reinemar@terra.com.br); *Alexis Dourado Guedes*, M.D. (dr.alexis@uol.com.br), Odelisa Silva de Mattos, M.D. (odelisam@yahoo.com.br); **Federal University of Maranhão**: Manuel Faria*, M.D. (mfaria@inlab.com.br), *Rossana Azulay*, M.D. (rossanaendocrino@gmail.com); **Diabetes and Hypertension Center of Ceará**: Adriana Costa e Forti*, M.D. (adrianaforti@uol.com.br), *Cristina Figueiredo Sampaio Façanha*, M.D. (crisffacanha@hotmail.com); **Federal University of Ceará**: Renan Montenegro Junior*, M.D. (renanjr@ufc.br), *Ana Paula Montenegro*, M.D. (clinicarenanmontenegro@hotmail.com); **Federal University of Sergipe**: Naira Horta Melo*, M.D. (nhmelo@gmail.com), *Karla Freire Rezende*, M.D. (kfr@infonet.com.br); **Federal University Hospital of Campina Grande, Paraíba**: Alberto Ramos*, M.D. (ajsr@uol.com.br); **Federal University Hospital of Pará**: João Soares Felício*, M.D. (felicio.bel@terra.com.br), *Flavia Marques Santos*, M.D. (drafms@bol.com.br); **Getúlio Vargas University Hospital of Amazonas, Adriano Jorge Hospital**: Deborah Laredo Jezini*, M.D. (dljezini@hotmail.com).

## Abbreviations

T1D: Type 1 diabetes; MNT: Medical nutrition therapy; BDS: Brazilian Diabetes Society; NBHCS: National Brazilian Health Care System; CV: Cardiovascular; HbA1c: Glycated hemoglobin; FPG: Fasting plasma glucose; LDL: Low density lipoprotein; HDL: High-density lipoprotein; BrazDiab1SG: Brazilian Type 1 Diabetes Study Group; BMI: Body mass index; NGSP: National Glycohemoglobin Standardization Program; IBGE: Brazilian Institute of Geography and Statistics; CI: Confidence interval; CT: Conventional therapy; IT: Intensive therapy; CSII: Continuous subcutaneous insulin infusion; SMBG: Self-monitoring of blood glucose.

## Competing interests

All the authors declare they do not have any conflict of interest.

## Authors’ contributions

KAKD, MBG and CAN contributed equally analyzing the data and writing the manuscript. MBG has full access to all study data and had the responsibility for the submission. All authors read and approved the final manuscript.
